# Supplementary material for: Anticipating Leucovorin Rescue Therapy in Patients with Osteosarcoma through Methotrexate Population Pharmacokinetic Model
Source: Pharmaceutics. 2024 Sep 6;16(9):1180. doi: 10.3390/pharmaceutics16091180 (PMC11434990; doi:10.3390/pharmaceutics16091180)
Supplement: Supplementary file 1 [file pharmaceutics-16-01180-s001.zip › pharmaceutics-3108328-supplementary.pdf]

Anticipating Leucovorin Rescue Therapy in Patients with Osteosarcoma through Methotrexate Population Pharmacokinetic Model

Laura Ben Olivo, Pricilla de Oliveira Henz, Sophia Wermann, Bruna Bernar Dias, Gabriel Osorio Porto, Amanda Valle Pinhatti, Manoela Domingues Martins, Lauro José Gregianin, Teresa Dalla Costa, Bibiana Verlindo de Araújo *

Supplemental Material

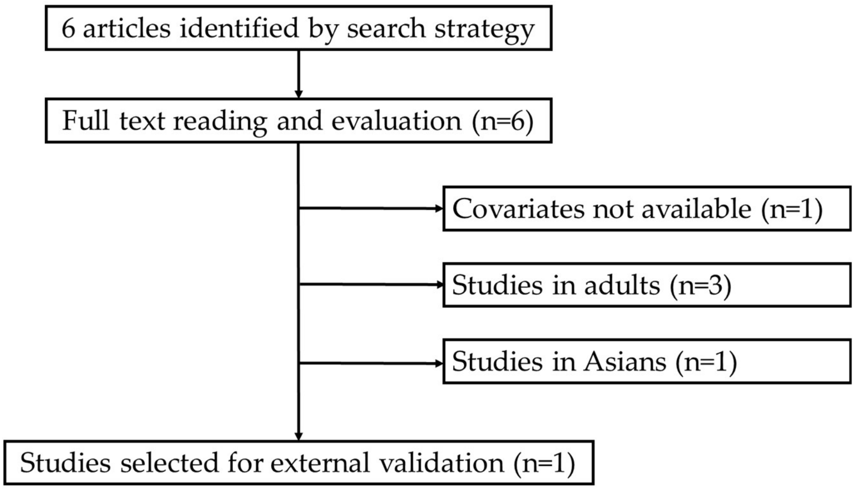

Figure S1: Flow diagram of the strategy used in the literature search.

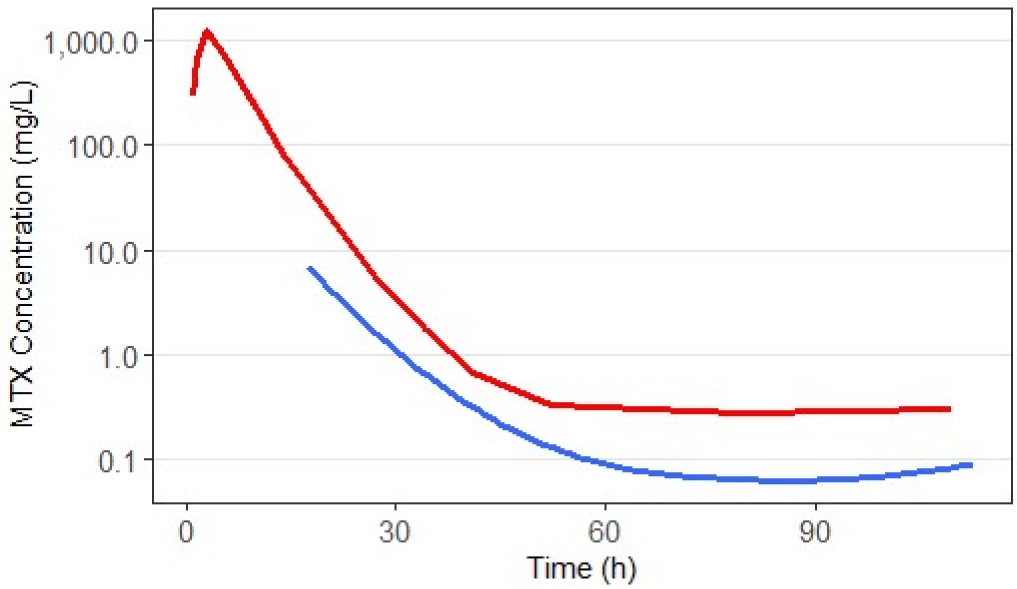

Figure S2: Mean MTX plasma concentration versus time of Spanish (red line) and Brazilian (blue line) pediatric patients with osteosarcoma.

Table S1: Covariate model development.

	Description	OFV	Δ OFV
Foward Inclusion			
1	Base model	-1513.84	-
2	Model 1 + θ_{Scr}	-1537.11	-23.272
3	Model 2 + θ_{BSA}	-1543.87	-6.755
Backward Elimination			
4	Model 3 - θ_{Scr}	-1532.96	10.913
5	Model 5 - θ_{BSA}	-1537.11	6.755
